# Early Exposure to Cardiac Treatment and Distress Among Patients and Their Caregiving Partners

**DOI:** 10.3389/fpsyg.2020.00141

**Published:** 2020-02-12

**Authors:** Talea Cornelius, Noa Vilchinsky, Keren Fait, Shlomi Matetzky, Hanoch Hod

**Affiliations:** ^1^Center for Behavioral Cardiovascular Health, Columbia University Irving Medical Center, New York, NY, United States; ^2^Department of Psychology, Bar-Ilan University, Ramat-Gan, Israel; ^3^Intensive Cardiac Care Unit, Leviev Heart Institute, Sheba Medical Center, Tel-Hashomer, Ramat Gan, Israel; ^4^The Sackler Faculty of Medicine, Tel-Aviv University, Tel-Aviv, Israel

**Keywords:** acute cardiac event, caregivers, acute care, anxiety, posttraumatic stress, couples

## Abstract

**Background:** The experience of an acute coronary event (ACE), including early care and evaluation, can be a distressing and traumatic experience for patients and their romantic partners, who also act as caregivers. We hypothesized that, among partners who were present during the ACE, those who were also present during (1) transportation to the hospital and (2) initial medical treatment would experience greater (a) anxiety early post-event and (b) posttraumatic stress symptoms (PSS) related to the event 4 months later. The associations between partner presence with patient anxiety and PSS were also explored.

**Methods:** Participants were ACE patients and their partners recruited between March 2015 and December 2016 from the Intensive Cardiac Care Unit (ICCU) of the Sheba Medical Center in Israel (*N* = 143; all patients were males and partners were females). Partners self-reported whether or not they were present during the cardiac event, the hospital drive, and initial care. Patients and partners self-reported anxiety in-hospital and PSS, keyed to the ACE, an average of 4 months later. Data were analyzed using General Estimating Equations (GEE) and Multilevel Modeling.

**Results:** Neither patient anxiety nor PSS differed according to partner presence during the drive to the hospital. In contrast, partners had higher anxiety when they were not present at all (difference = 3.65, *p* = 0.019) and when present during the event and during the drive (difference = 2.93, *p* = 0.029) as compared to when they were present for the event but not for the drive. Partners who were present during the event, but not the drive, had lower PSS than those who were present for both the event and the drive (difference = −4.64, *p* = 0.026).

**Conclusions:** Partners who accompany patients on the drive to the hospital may inadvertently put themselves at risk for greater distress following their loved one’s cardiac event. Future research should enroll couples in an acute care context to inform couple-targeted tailored interventions to reduce distress in patients and their caregiving partners.

## Early Exposure to Cardiac Treatment and Distress Among Patients and Their Partners

Romantic partners are a key source of support, and high-quality romantic relationships are linked to a wide range of positive health outcomes (Robles and Kiecolt-Glaser, 2003; King and Reis, 2012; Robles et al., 2014). One context in which social support is thought to be beneficial is emergency health situations (United Hospital Fund, 2012), for example, having a support person present during a life-threatening event such as acute coronary event (e.g., acute coronary syndrome, cardiac arrest). Yet, recent research suggests that close others in an emergency context may actually cause distress in patients (Cornelius et al., 2019a), and some who accompany patients to the emergency department (ED) are ill-equipped to provide social support (Homma et al., 2016). A possible explanation for this may be that an acute coronary event is also incredibly distressing to partners. Indeed, the negative psychological impact on partners can be profound (Fait et al., 2017; Vilchinsky, 2017). Scarce research exists on the impact of partner presence during early emergency care on patients, and even less addresses the impact of presence during early care on partner distress. The present study addressed this gap by examining the associations of partner presence during (1) transportation to the hospital and (2) initial medical treatment with (a) patient and partner anxiety early post-event and (b) posttraumatic stress symptoms (PSS) related to the event 4 months later in a sample of patients experiencing an acute coronary event and their partners.

The experience of an acute coronary event, including early care and evaluation, can be a distressing and traumatic experience for patients and romantic partners alike. A new line of research suggests that close others in the ED, such as a spouse or romantic partner, may actually cause patients distress when contrasted to patients who arrive with non-close others (e.g., a neighbor) or alone (Cornelius et al., 2019a). Although the reasons for this remain unclear, partner distress may exacerbate the already-stressful emergency care environment. Patients’ romantic partners are also greatly impacted by the acute coronary event (Mahrer-Imhof et al., 2007; Dalteg et al., 2011). Thus, romantic partners also likely feel threatened and anxious during the early uncertainty of emergency care. Not only might this undermine the ability of distressed partners to provide positive social support to patients (Cornelius et al., 2019b), but distress in one member of a couple can elicit further distress in the other. Indeed, exposure to the suffering of a spouse increases physiological reactivity (e.g., increased blood pressure) (Monin et al., 2010), and patients and partners can spread negative emotions to each other *via* a process of “emotional contagion” (Gump and Kulik, 1997; West et al., 2017). Critically, distress (depression in particular) is an independent risk factor for incident cardiovascular diseases (Van der Kooy et al., 2007; Whooley et al., 2008; Ho et al., 2010, 2018).

Presence during an acute coronary event, including both the event itself and exposure to early care, can be conceptualized as a triggering event for distress in romantic partners. An acute coronary event is an “exposure” that can lead to PSS in patients and in their partners (Edmondson et al., 2012; Edmondson, 2014; Fait et al., 2017; Vilchinsky, 2017), and characteristics of the early emergency care environment (e.g., ED crowding) can also contribute to elevated PSS (Chang et al., 2016). Whereas patients are necessarily exposed to the cardiac event and the acute care environment, the partner’s level of exposure varies depending on whether or not they were present during the event itself, and whether or not they were also exposed to early evaluation and care (i.e., from the point where the decision was made that this was a serious event and emergency treatment was needed). It is possible that partners who are exposed to the triggering event and also attempt to be present as a source of support, with the best intentions of caregiving, may simultaneously put themselves at risk for greater traumatization *via* increased exposure to the stress of the acute care environment.

The present study is one of the first to examine the impact of event exposure and early treatment exposure on distress in both patients experiencing an acute cardiac event (ACE) and their partners. The primary question is thus: *what is the association between partner presence during an acute coronary event and early medical treatment with distress* (i.e., *anxiety and PSS*) *related to the ACE in both patients and partners?* Drawing on our previous work, we hypothesized that, among partners who were exposed to the cardiac event (i.e., who were exposed to the cardiac event at onset), presence also during (i.e., exposure to) (1) the drive to the hospital and (2) initial treatment in the hospital would be associated with (a) higher anxiety early post-treatment in both patients and their partners and (b) greater PSS in partners approximately 4 months post-ACE. We hypothesized the link between partner presence and partner PSS because presence can be seen as similar to trauma exposure (Vilchinsky, 2017; Vilchinsky and Dekel, 2018). However, since in a prior study, we did not find evidence for a direct effect of close others on distress distal to an acute coronary syndrome (ACS) in patients (Cornelius et al., 2019a), we refrained from hypothesizing the same link for patients. We anticipated that partners who were not at all exposed to the event would be the least distressed.

## Methods

### Participants

Participants were drawn from a larger, longitudinal study examining the etiology and progression of ACE-induced PSS in ACE patients and their partners. Patients were diagnosed with an acute coronary event [i.e., ACS, unstable angina (UA), myocardial infarction (MI), cardiac arrest (CA)] and reported being in a committed romantic relationship. The index event did not have to be a first acute cardiac event. Patients with non-cardiac diagnoses (aside from cardiac risk factors, e.g., diabetes, high blood pressure, hypercholesterolemia) were ineligible. Other exclusion criteria included elective hospitalization, cognitive, physical, or language difficulties that precluded interviews, coronary artery bypass graft (CABG) surgery during hospitalization, age greater than 85, death during hospitalization, tourists, and guardianship. Both patients and partner had to agree in order to participate in the study. Partners were also excluded if they were of an age greater than 85, or had cognitive, physical, or language difficulties that precluded interviews.

### Procedure

Participants were recruited from the Intensive Cardiac Care Unit (ICCU) of the Sheba Medical Center, the largest medical center in Israel. Eligible patients were identified *via* electronic medical record and approached in-hospital to gauge interest in participating. The research team then contacted the partners of patients who were interested in participating. Couples in which both members were eligible and agreed to participate then completed informed consent and baseline questionnaires during hospitalization (*via* self-report or interview). Beginning no earlier than 2 months post-discharge (time 2), couples were contacted by phone to complete a second interview at home. A time 3 follow-up was completed on average 8 months after time 2 (data not included in this analysis). Data collection occurred between March 2015 (time 1), November 2017 (time 2), and March 2018 (time 3). All procedures were approved by the Sheba Medical Center institutional review board.

### Measures

#### Partner Presence

Partners self-reported whether or not they were present during the hospital drive and during initial care with two questions (*1*, “yes,” or *0*, “no”): “Did you accompany your partner to the hospital (by ambulance or private vehicle)?” and “Were you present during the initial treatment in the emergency room/department?”

#### Anxiety

Patients and partners self-reported anxiety following the ACE at baseline using the Hospital Anxiety and Depression Scale (HADS), which has been validated among Hebrew speakers (Snaith, 2003). Anxiety is assessed *via* 7 items, scored from 0, “not at all,” to 3, “very often,” and summed to form a total score. Reliability scores according to Cronbach’s alpha were 0.789 and 0.893 for patients and partners, respectively.

#### Posttraumatic Stress Symptoms

PSS were self-reported by patients and partners at time 2 post-ACE using the Posttraumatic Diagnostic Scale for DSM-5 (PDS-5), keyed to the ACE. The PDS-5 is a valid and reliable scale, and is scored by summing 20 items that assess DSM-5 symptom clusters of intrusion, avoidance, changes in mood and cognition, and arousal and hyperactivity (Foa et al., 2016). Response options range from 0, “not at all,” to 4, “6 times and above/to an extreme extent” (e.g., “disturbing and unwanted memories of the event”). A cutoff of 28 or greater can be used to identify a positive screen for significant PSS (Foa et al., 2016). Reliability scores at time 2 according to Cronbach’s alpha were 0.887 and 0.933 for patients and partners, respectively.

#### Covariates

Covariates were selected *a priori*, and included: (1) self-reported age (patients only); (2) income, rated on a scale from 1, “significantly above average,” to 5, “significantly below average”; (3) illness severity according to the echocardiogram, rated from 1, “none,” to 4, “severe” (scored by a senior cardiologist, blind to study hypotheses); and (4) whether the couple participated at follow-up.

### Data Analysis Strategy

Data from couples are interdependent, violating traditional regression assumptions and necessitating modeling strategies that can account for this relationship (Kenny et al., 2006). Preliminary analyses were conducted using General Estimating Equations (GEE) (Hardin and Hilbe, 2012) in SPSS v.25 (2017). Primary analyses were conducted using Multilevel Modeling (MLM; Hox, 2013) in Mplus V.8.0 statistical package (Muthén and Muthén, 1998–2017).

Analyses testing the effect of partner’s presence on anxiety and PSS were conducted in two stages. For anxiety and PSS, we tested a series of models separately for each presence variable (i.e., presence during the drive, presence during initial care). In Model 1, we tested main effects of the independent variables: role (either patient or partner) and partner presence (partner was not present during the event at all; partner was present during the event and during the drive/initial care; and partner was present during the event but not during the drive/initial care)^1^. In Model 2, the multiplicative interaction between role and partner presence was added. If significant effects of partner presence were uncovered in these preliminary GEE models, the same two models were specified (i.e., Model 1 and Model 2) using multilevel modeling and adjusting for pre-specified covariates in Mplus V.8.0 statistical package (Muthén and Muthén, 1998–2017).

Missing data were incorporated using Full Information Maximum Likelihood (FIML; Enders, 2010; Bar, 2017), which allows all participants providing any data to be included in the analysis. This approach was valid; a preliminary test of the missing values yielded *p* = 0.673; thus, we did not reject the null hypothesis [i.e., that data were missing completely at random (MCAR)] (Little, 1988). We additionally included a dummy-coded control variable (“Time 2- no missing”).

## Results

### Characteristics of the Sample

Of 461 potentially eligible patients, 81 (17.57%) were discharged before the research team was able to contact them and 38 (8.24%) had partners who were unavailable for interview. Of the remaining 342, 156 (45.61%) couples agreed to participate and completed baseline questionnaires. Of these couples, only 13 dyads (8.3%) consisted of female patient/male partner pairs. We therefore conducted *t*-tests to assess gender differences in anxiety and PSS within each role (patient/partner). Results revealed that female patients’ anxiety levels (*M* = 7.30, SD = 5.73) were significantly higher than those of male patients (*M* = 4.42, SD = 4.10; *t* = 2.337, df = 154; *p* = 0.021). Conversely, female partners’ anxiety levels (*M* = 5.00, SD = 3.96) were significantly lower than male partners’ anxiety (*M* = 8.42, SD = 5.32; *t* = 2.261, df = 154; *p* = 0.025). The same trends were detected for PSS (though not statistically significant). Because there were not enough female patients to detect significant gender by role interactions, we excluded these 13 dyads from further analysis and focused instead on the 143 male patient/female partner dyads.

Of these 143 dyads, 106 provided complete data (74%), and 37 dyads (26%) provided responses for the first interview only. Mean patient age was 56.345 (SD = 10.944) and mean income level was 3.156 (SD = 1.105; 3 indicated “average” income on this scale). Mean illness severity was 2.077 per echocardiogram (SD = 1.095). The majority of partners were present for the hospital drive (85, 60%), about a third of the sample were not present at all during the cardiac event (40, 28%), and only few partners were present during the event but did not escort the patient to the hospital (18, 12%). Most partners (82, 57%) were present during patients’ initial care at the hospital.

#### Preliminary Analyses

Unconditional models showed relatively high intraclass correlation coefficients (ICC > 0.05; ICC_anxiety_ = 0.097, ICC_PSS_ = 0.185), indicating significant interdependence in the data. For anxiety, GEE followed by *post hoc* pairwise comparisons with Bonferroni correction revealed a significant interaction effect between role (patient/spouse) and partner presence during the cardiac event and drive to the hospital (Wald = 9.00, df = 2, *p* = 0.011). No significant differences as a function of partners’ presence emerged for patients’ anxiety levels; however, *partners’* anxiety was significantly higher if she was not present during the event or was present during the event and the drive to the hospital, compared to being present during the event but not the drive. No significant interaction between role and partner presence, as defined by presence during initial care, emerged for anxiety (Wald = 2.15, df = 2, *p* = 0.340).

For PSS, a marginally significant interaction emerged between role and partner presence during the drive to the hospital (Wald = 4.99, df = 2, *p* = 0.082), but the interaction between role and presence during initial care was not significant (Wald = 0.09, df = 2, *p* = 0.995). Thus, multilevel analyses were conducted only to examine the effects of partner presence during the drive to the hospital on patient and partner distress (and not during initial care). Means and standard deviations of anxiety and PSS for patients and partners, stratified by partner presence during the cardiac event and drive to the hospital, are in Table 1.

**Table 1:** Means and standard deviations of anxiety and PSS for patients and partners by context of presence during the cardiac event.

Partner presence during the cardiac event		Anxiety at hospitalization			PSS at follow-up		
		*n*_dyads_	*M*	SD	*n*_dyads_	*M*	SD
Not present at all	Patient	40	4.525	3.789	26	6.461	9.521
	Partner		9.600	5.999		8.878	9.478
Present also during drive	Patient	85	4.259	4.044	66	6.394	8.973
	Partner		8.494	4.775		9.506	12.598
Present but not during drive	Patient	18	5.000	5.156	14	9.595	15.582
	Partner		5.500	5.415		4.143	5.722

*PSS, Posttraumatic stress symptoms*.

#### Primary Analyses

Full results for multilevel models predicting anxiety and PSS, including all covariates, are detailed in Tables 2, 3, respectively. In main effects models (Model 1), partners exhibited significantly higher anxiety than patients, *B* = 4.00, se = 1.76, *p* < 0.001. However, there was no difference between patients and partners for PSS, *B* = 1.81, se = 1.32, *p* = 0.17. No significant main effects emerged of partner presence during the cardiac event or during the drive to the hospital on either anxiety or PSS, *p*’s > 0.05.

**Table 2:** The main and interactive effects for anxiety as the outcome measure.

	Model 1		Model 2	
	Estimates	S.E.	Estimates	S.E.
**Level 2 (dyadic level)**				
Patients’ age	−0.06^*^	0.03	−0.06^*^	0.03
Patients’ EF severity	0.62^*^	0.25	0.62^*^	0.25
Family income	−0.82^**^	0.31	−0.82^**^	0.31
Partner presence (0 vs. 2)	1.21	1.00	−1.08	1.19
Partner presence (1 vs. 2)	1.06	0.80	−0.81	1.10
Time 2 – no missing	−1.40	0.74	−1.40	0.74
Residual variances	4.16^*^	1.76	4.62^**^	1.76
**Level 1 (individual level)**				
Role	4.00^***^	(0.47)	0.50	1.75
Role * partner presence (0 vs. 2)	—	—	4.58^*^	1.91
Role * partner presence (1 vs. 2)	—	—	3.74^*^	1.84
Residual variances	16.03^***^	(1.95)	15.08^***^	2.03
CFI	1.00		1.00	
TLI	1.00		1.00	
RMSEA	0.000		0.000	
SRMR	0.058		0.058	
Chi-square	8.75		8.75	
df	10.00		10.00	
*p*	0.56		0.56	
Intraclass correlation (ICC)	0.097			

*CFI, Confirmatory Fit Index; TLI, Tucker-Lewis Index; RMSEA, root mean square error of approximation; SRMR, standardized root mean square residual*.

Role: (1) = partner, (0) = patient. Partner presence: [(0) partner was not present during the event at all; (1) partner was present during the event and escorted the patient to the hospital; and (2) partner was present during the event but did not escort the patient to the hospital]. Level 1 represents the variables characterizing each participant, whereas Level 2 represents the variables characterizing each couple. Model 1 represents the main effects in both levels and model 2 adds the interactions measured at Level 1;

* p < 0.05;

** p < 0.01;

*** *p < 0.001*.

**Table 3:** The main and interactive effects for PSS as the outcome measure.

	Model 1		Model 2	
	Estimates	S.E.	Estimates	S.E.
**Level 2 (dyadic level)**				
Patients’ age	−0.01	0.07	−0.01	0.07
Patients’ EF severity	0.77	0.85	0.77	0.85
Family income	−1.42~	0.76	−1.42	0.76
Partner presence (0 vs. 2)	−0.38	2.80	−4.31	4.31
Partner presence (1 vs. 2)	0.36	2.66	−3.92	4.13
Residual variances	19.20^*^	9.49	20.89^*^	9.96
**Level 1 (individual level)**				
Role	1.81	1.32	−5.45	3.39
Role * Partner presence (0 vs. 2)	—	—	7.87^*^	3.92
Role * Partner Presence (1 vs. 2)	—	—	8.56^*^	3.83
Residual variances	91.38^***^	24.63	87.58^***^	23.66
CFI	1.00		1.00	1.00
TLI	1.00		1.00	1.00
RMSEA	0.000		0.000	0.000
SRMR_within_	0.002		0.001	0.002
SRMR_between_	0.044		0.054	0.044
Chi-square	6.94		6.95	6.94
df	7		7	7
*p*	0.44		0.43	0.44
Intraclass correlation (ICC)	0.185			0.185

*CFI, Confirmatory Fit Index; TLI, Tucker-Lewis Index; RMSEA, root mean square error of approximation; SRMR, standardized root mean square residual*.

*Role: (1) = partner, (0) = patient. Partner presence: [(0) partner was not present during the event at all; (1) partner was present during the event and escorted the patient to the hospital; and (2) partner was present during the event but did not escort the patient to the hospital]. Level 1 represents the variables characterizing each participant, whereas Level 2 represents the variables characterizing each couple. Model 1 represents the main effects in both levels and model 2 adds the interactions measured at Level 1*.

* p < 0.05;

** p < 0.01;

*** *p < 0.001*.

**Figure 1:**
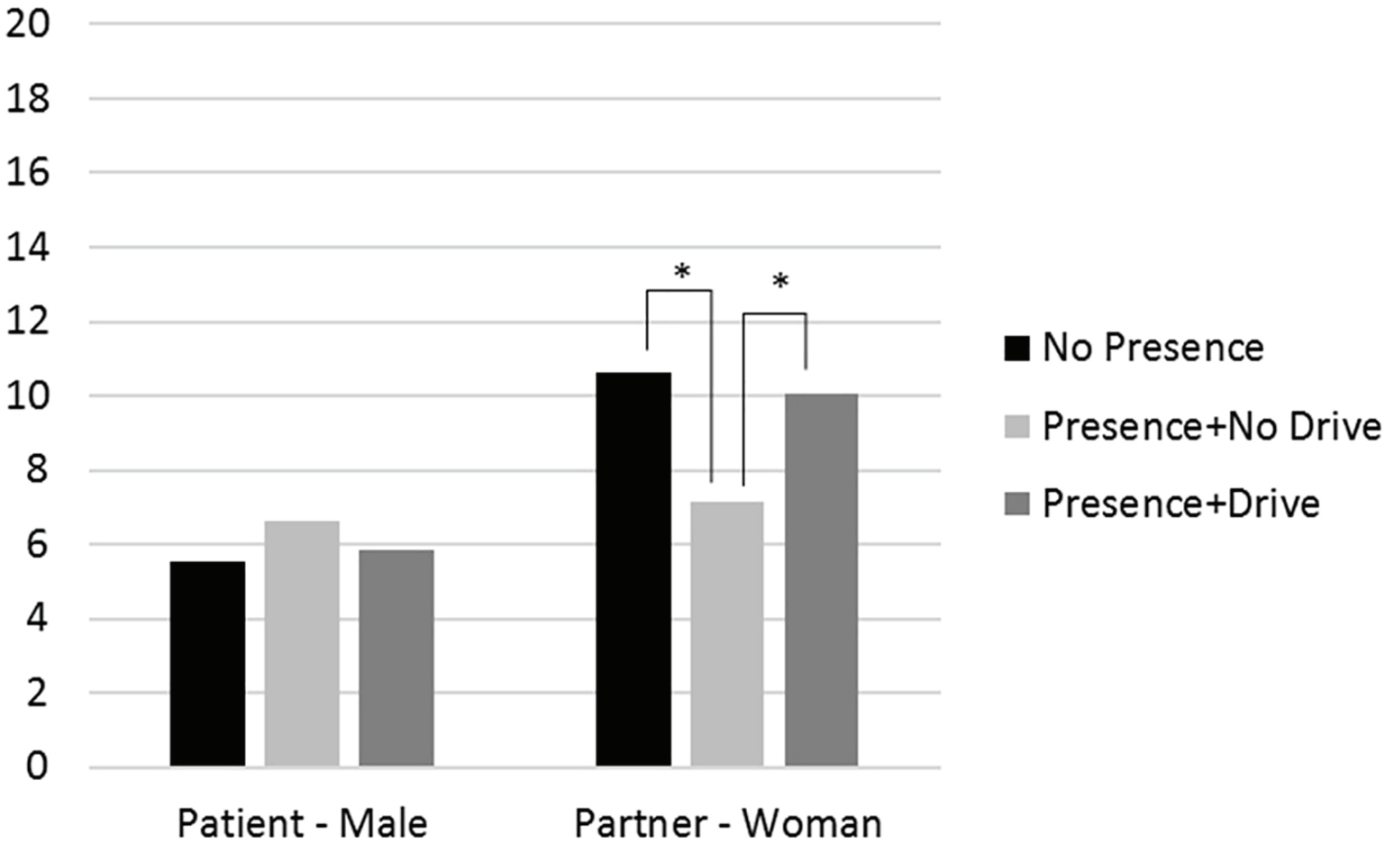
The effects of partners’ presence during the drive to the hospital on patients’ and partners’ anxiety levels as measured during patients’ hospitalization for an average respondent (i.e., average age, income, and illness severity). Note: * = *p <* 0.05.

**Figure 2:**
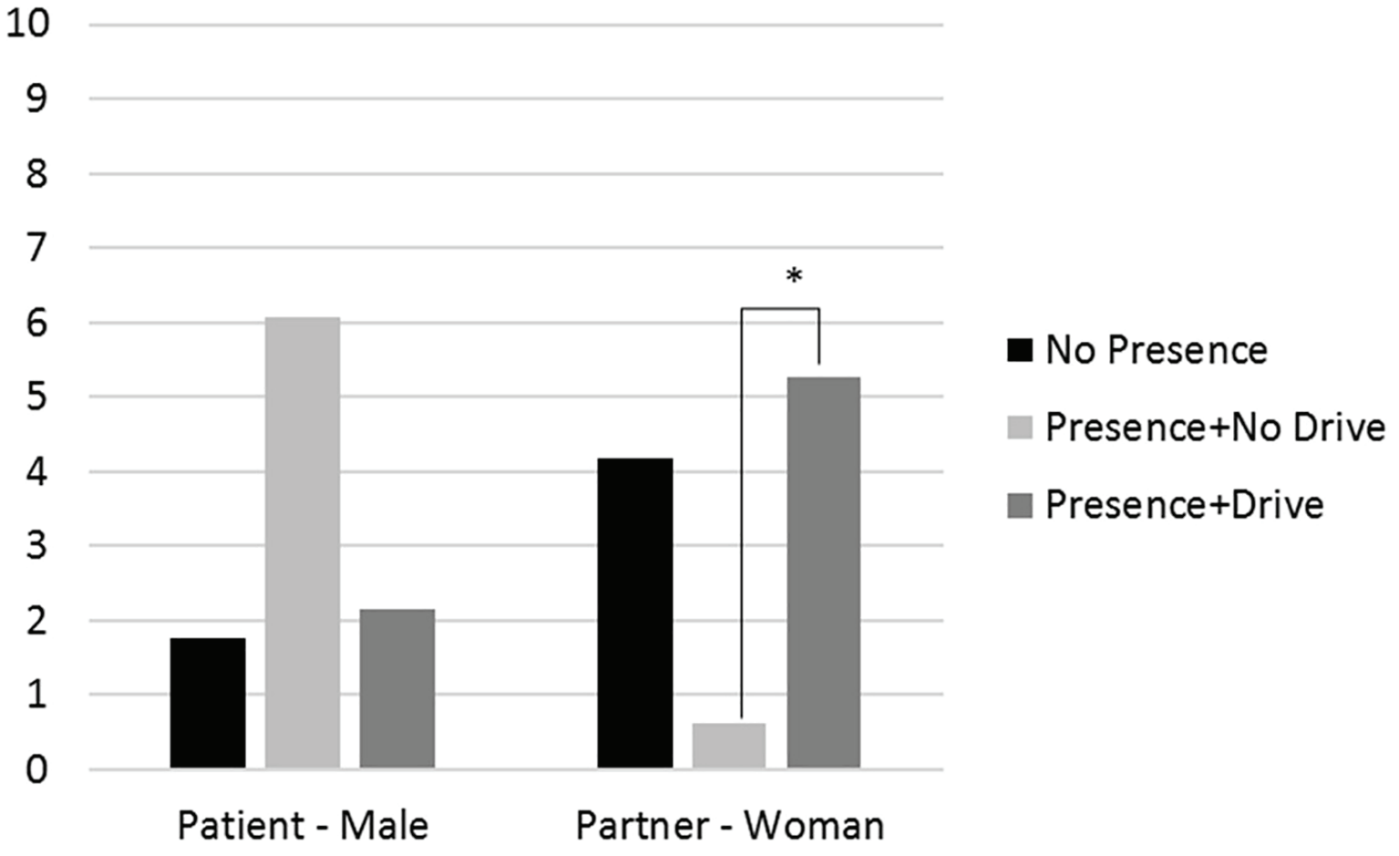
The effects of partners’ presence during the drive to the hospital on patients’ and partners’ PSS levels as measured at follow up for an average respondent (i.e., average age, income, and illness severity). Note: * = *p <* 0.05.

Including the multiplicative term of role with partner presence (Model 2) revealed a significant interaction effect predicting both anxiety and PSS (*p’*s < 0.05; see Figures 1, 2). Follow-up analyses showed that patients reported lower anxiety than partners when partners were present during the event and the drive to the hospital (difference = −4.24, *p* < 0.001) and when partners were not present at all during the event (difference = −5.08, *p* < 0.001), but not when partners were present during the event but did not accompany the patients to the hospital (difference = −0.50, *p* = 0.78). For patients, anxiety was not different depending on partner presence. In contrast, partners had higher anxiety when they were not present at all (difference = 3.65, *p* = 0.019) and when present during the event and during the drive (difference = 2.93, *p* = 0.029), as compared to when they were present for the event but not for the drive. For PSS, the only significant comparison was that partners who were present during the event, but not the drive, had lower PSS than those who were present for both the event and the drive (difference = −4.64, *p* = 0.026).

#### Sensitivity Analyses

Similar results were obtained when models were estimated among participants providing full data only (*n* = 106 dyads) and when the dummy code indicating time 2 participation was dropped from the anxiety analysis. Including transportation type (ambulance v. private car) as a covariate did not alter study results.

## Discussion

The present study is one of the first to examine the impact of partner presence during early care for a life-threatening health event on psychological outcomes in patients experiencing an acute cardiac event and their caregiving partners. Although people are encouraged to have a supportive other present in such situations (United Hospital Fund, 2012), results suggest that this may not always be beneficial. In this study, there was no significant benefit to patients when partners were present during the event or during transportation to the hospital on either anxiety or on PSS, and partner presence during initial care at the ICCU also did not appear to be related to patient distress (in preliminary tests). Furthermore, partners who were present during the cardiac event and also accompanied patients during transportation to the ED were more anxious and had greater PSS than those who were not present during the drive. Partners who were not present at all were also more anxious than those who were present during the cardiac event, but not during the drive.

Emerging research has begun to illuminate the potential for negative psychological effects in patients who have a close other, such as a spouse or romantic partner, present during early emergency care. For example, one study conducted at an urban medical center in the United States found that patients in the ED with close others reported feeling more threatened, helpless, and vulnerable when asked to recall their ED experience only a few days later (Cornelius et al., 2019a). There was no impact of the presence of a partner on patient anxiety in the current study, however. This could be due to differences in the way psychological distress was measured. Feeling threatened is tied specifically to the acute care experience; conversely, anxiety (as assessed in this study) is a more global construct, which could have precluded the detection of negative psychological effects in patients specific to the acute care environment (e.g., patient distress specifically during the drive to the hospital).

In contrast, in the current study, partner distress was related to presence during the cardiac event and during the drive to the hospital. Those partners who accompanied the patients during the drive to the hospital tended to be more anxious and have greater PSS than those who witnessed the emergence of symptoms, but did not accompany the patient to the ICCU. It may be that those who accompanied the patient during the drive were exposed to additional frightening sights, such as early uncertainty about the nature of the event, or viewing lifesaving intrusive efforts taking place in the ambulance. Indeed, these early and uncertain moments may be particularly distressing. Conversely, it may be that partners who are most prone to distress are likely to accompany patients to the hospital. Counter to our hypotheses, those partners who were not at all present during the cardiac event were also more anxious than those who were present but did not join the patient on the drive to the ICCU. Lack of information and hearing about the cardiac event *via* a third party could contribute to feeling out of control, helpless, or anxious.

It is unclear why presence during the drive to the hospital, but not during initial care, was related to anxiety in spouses. It may be that the earliest exposure—when patients and spouses decide that it is necessary to go to the ED but have no information yet—is the most detrimental due to the largest amount of uncertainty, whereas early hospital care includes diagnosis and more information about patient prognosis that could alleviate some of this distress. Indeed, it is specifically anxiety that is stoked by uncertainty prior to receiving bad news (e.g., about a health event), but other emotions (e.g., sadness) are more pronounced once that information has been received (Sweeny et al., 2009; Sweeny and Falkenstein, 2015). Because early anxiety and distress predict PSS, distress that occurs and develops during a time when the hospital care team is present, this presents an incredible and unique opportunity to address the development of PSS *during* traumatic exposure, something that is unheard of within other contexts (e.g., combat, assault, etc.). Indeed, some intervention work suggests that providing family members with additional information may alleviate distress (Goldfarb et al., 2017). To inform such interventions, future studies should explore the evolution of distress in patients and partners over the course of acute care in relation to the amount of information received, such as anxiety pre- and post-diagnosis, treatment recommendations, and discharge planning.

### Limitations

Results should be considered in light of a number of limitations. The sample was recruited from one medical center in Israel, and may not generalize to other populations. Analyses should also be replicated in larger and more diverse samples. The effects uncovered in the present study were not large, and may have been further attenuated by distal assessments of distress not specifically tied to the care experience. Partner presence may also have been determined by third variables. Specifically, it may be that those partners who witnessed the cardiac but did not escort the patient during the drive were less anxiety prone, had a general lack of interest in patients’ health, or were confident that the situation was not that dire.

Still, although preliminary, this study presents some of the first data beginning to unpack the effects of early acute care on psychological distress simultaneously in patients and their partners.

## Conclusions

Although social support from a romantic partner is often thought to be beneficial (Robles and Kiecolt-Glaser, 2003; King and Reis, 2012; Robles et al., 2014) and it is generally recommended to have a support person present during emergency care situations (United Hospital Fund, 2012), emerging research suggests that close support partners may actually increase patient distress in the ED (Homma et al., 2016; Cornelius et al., 2019a,b) or fail to alleviate distress, as in the present study. Furthermore, this study suggests that partners who accompany patients on the drive to the hospital may inadvertently put themselves at risk for greater anxiety following their loved one’s cardiac event. There is a need for future research in both patients and their partners in an acute care context to inform couple-targeted tailored interventions to reduce distress in patients and their partners.

## Data Availability Statement

The raw data supporting the conclusions of this article will be made available by the authors, without undue reservation, to any qualified researcher.

## Ethics Statement

The studies involving human participants were reviewed and approved by the Sheba Medical Center ethics committee. The patients/participants provided their written informed consent to participate in this study.

## Author Contributions

TC contributed to analysis and interpretation of the findings, to drafting and writing the manuscript. NV contributed to conception and design of the study, to the acquisition, analysis, and interpretation of the findings, and to drafting and writing the manuscript. KF contributed to the conception and design of the study, and to the acquisition of the findings. SM contributed to data collection. HH contributed to data collection and interpretation of the findings. All authors contributed to manuscript revision, read and approved the submitted version.

### Conflict of Interest

The authors declare that the research was conducted in the absence of any commercial or financial relationships that could be construed as a potential conflict of interest.

## Footnotes

^1^No partners were present for the drive/during care but not during the cardiac event.
